# Stigma and burden of mental illness and their correlates among family caregivers of mentally ill patients

**DOI:** 10.1186/s42506-020-00059-6

**Published:** 2020-11-09

**Authors:** Omnya S. Ebrahim, Ghada S. T. Al-Attar, Romany H. Gabra, Doaa M. M. Osman

**Affiliations:** 1 Department of Community, Occupational and Environmental Medicine, Faculty of Medicine, Helwan University, Helwan, Egypt; 2 Department of Public Health & Community Medicine, Faculty of Medicine, Assiut University, Assiut, Egypt; 3 Department of Neuropsychiatry, Assiut University Hospital, Assiut University, Assiut, Egypt

**Keywords:** Stigma, Burden, Mental illness, Family caregivers, Egypt

## Abstract

**Background and objectives:**

Family caregivers play a curial role in supporting and caring for their mentally ill relatives. Their struggle for facing stigma and shouldering caregiving burden is marginalized, undervalued, and invisible to medical services. This study assessed the stigma and burden of mental illnesses, and their correlates among family caregivers of mentally ill patients.

**Methods:**

A cross-sectional study design was used to collect data from 425 main family caregivers of mentally ill patients at Assiut University Hospital. A structured interview questionnaire was designed to collect socio-demographic data of both patients and their caregivers. Stigma scale for caregivers of people with mental illness (CPMI) was used to assess the affiliate stigma, while the associative stigma was assessed by the explanatory model interview catalogue stigma scale (EMIC-Stigma scale). The caregivers’ burden was assessed using Zarit burden Interview, and Modified Attitude toward Mental Illness Questionnaire was used to assess caregivers’ knowledge and attitude towards mental illness.

**Results:**

Bipolar disorder (48%) and schizophrenia/other related psychotic disorders (42.8%) were the most common mental illnesses among the study patients. The mean scores of CPMI total scale, EMIC-Stigma scale, and Zarit Burden scale were 56.80 ± 7.99, 13.81 ± 5.42, and 55.20 ± 9.82, respectively. The significant correlates for affiliate stigma were being parents of patients (ß = 4.529, *p* < 0.001), having higher associate stigma (ß = 0.793, *p* < 0.001), and aggressive behavior of mentally ill patients (ß = 1.343, *p* = 0.038). The significant correlates for associate stigma of the study caregivers were being caregivers’ relatives other than parents (ß = 1.815, *p* = 0.006), having high affiliate stigma (ß = 0.431, *p* < 0.001), having poor knowledge and negative attitude towards mental illness (ß = − 0.158, *p* = 0.002), and aggressive behavior of mentally ill relatives (ß = 1.332, *p* = 0.005). The correlates for the high burden were being male (ß = 3.638, *p* = 0.006), non-educated caregiver (ß = 1.864, *p* = 0.045), having high affiliate stigma (ß = 0.467, *p* < 0.001), having high associative stigma (ß = 0.409, *p* < 0.001), having poor knowledge and negative attitude toward mental illness (ß = − 0.221, *p* = 0.021), seeking traditional healers and non-psychiatrist’s care from the start (ß = 2.378, *p* = 0.018), and caring after young mentally ill relatives (ß = − 0.136, *p* = 0.003).

**Conclusion:**

The studied caregivers suffered from stigma and a high level of burden. Psycho-educational programs directed toward family caregivers are highly recommended.

## Introduction

Mental illnesses are prevalent worldwide and in Egypt. The global prevalence of common mental disorders is approximately 1 in 5 adults (17.6%) [1]. A recent WHO meta-analysis estimated that the prevalence of mental disorders was 22.1% in emergency settings [2]. In Egypt, the National Survey of Mental Disorders estimated overall prevalence as 16.93% of the studied adult population [3].

The discrimination and stigma surrounding mental illnesses are widespread [4]. Stigma is defined as the prejudice, avoidance, rejection, and discrimination directed at people believed to have an illness, disorder, or other trait perceived to be undesirable [5]. Stigma in mental illness is a serious social problem that has a multitude of consequences on the individual concerned and his or her family [6].

Families are the mainstay of caregiving for persons with mental illnesses, especially in the Middle East [7]. Caregiving is a time-consuming responsibility, creating social, emotional, behavioral, and financial problems for the caregivers and causing various limitations on their personal life [8]. The continuous stress of caregiving adversely affects their physical and mental health. Moreover, it may affect their ability to care for their mentally ill relatives [9]. However, mental health professionals focus only on the index patient and the relatives’ needs and concerns are often ignored [10].

Few studies in Egypt, specifically in Upper Egypt [7] explored the stigma and burden among family caregivers of mentally ill patients. The current study assessed the stigma and burden and their correlates among family caregivers of mentally ill patients in Assiut governorate.

## Methods/experimental

### Study setting

The study was conducted in outpatient clinics and inpatient wards in the Neurology and Psychiatry Hospital affiliated to Assiut University. Assiut University is located in the capital city of Assiut Governorate, which is the largest city in Upper Egypt. The Neurology and Psychiatry Hospital serves patients from all governorates of Upper Egypt with a large working capacity [11].

### Study design and sample size

This study was a cross-sectional study targeting family caregivers of patients with mental disorders attending the previously mentioned setting. Data were collected during the period from 11 March to 14 August 2017. The total sample size was 425 caregivers. The sample size was estimated using the EPI info statistical package version 7.2.01 assuming that the proportion of stigma among caregivers is 0.5, a 95% confidence level, 5% margin of error, and 10% non-response rate.

### Study participants

The family caregivers of mentally ill patients who fulfilled the following criteria were included in the study:

- Caregivers aged ≥ 21 years.
- Must be intimately involved in the care of the patient for ≥ 1 year (i.e., looking after the daily needs, supervising the medications, bringing the patient to the hospital, staying with the patient during the inpatient stay, and maintaining liaison with the hospital staff).
- Caring for patients who met the principal diagnosis of any mental disorder according to the Diagnostic and Statistical Manual of Mental Disorders-Fifth Edition (DSM-5) criteria for ≥ 1 year. The diagnosis of mental illness was obtained from the patients’ records.

### Data collection tools

Data were collected by interviewing the caregivers using a structured predesigned questionnaire. The questionnaire included six parts:

1. Socio-demographic characteristics of both caregivers (age, gender, occupation religion, marital status, educational level, residence, and relationship with the patient) and the patients (age, gender, marital status, education, and source of patient’s income).
2. Mental illness history [duration of mental illness, aggressive behavior (injuring or threatening to injure anyone), suicide, times of inpatient psychiatric hospital admission in the past 6 months], and seeking help from non-psychiatric physicians and traditional healers before hospital attendance [12].
3. Stigma scale for caregivers of people with mental illness (CPMI) [13]: CPMI measures caregivers’ affiliate stigma (self-internalization of stigma) and has three components: affective (7 items), cognitive (7 items), and behavioral (8 items) components. The scale is composed of 22 items on a 4-point scale ranging from strongly disagree (1) to strongly agree (4). The scale ranges from 1 to 88, a higher score indicating a higher level of affiliate stigma.
4. Explanatory model interview catalogue-stigma scale (EMIC-Stigma scale) [14]**:** EMIC-stigma scale assesses caregivers’ associative stigma. Associate stigma is a process in which the caregivers are stigmatized from the public by their association with mentally ill individuals [15]. It has 15 questions, with four answer options [yes (3), possibly (2), uncertain (1), and no (0)]. The scale ranges from zero to 45, with a higher score indicating higher associate stigma.
5. Modified attitude toward mental illness questionnaire (ATMIQ) [16]. The Modified ATMIQ has 17 items that measures mental health literacy of the participants regarding causes of any mental illness (2 items), knowledge of people with mental illness (3 items), attitude toward people with mental illness (6 items), and management of people with mental illness (6 items). It is a 3-point Likert scale [agree (0), neutral (1), disagree (2)] that ranges from 0 to 34.

The three previously mentioned scales were translated from English to Arabic by the researchers, and then they were revised by a psychologist and a linguistic consultant and were subjected to reliability testing. Cronbach’s alpha of the CPMI scale was 0.87, Cronbach’s alpha of its affective, cognitive, and behavioral subscales was 0.81, 0.85, and 0.62, respectively, and Cronbach’s alpha of EMIC-stigma scale was 0.69. Cronbach’s alpha of modified ATMIQ was 0.68.

6- Arabic version of Zarit Burden Interview [17]; Zarit Burden Interview was originally designed to reflect the burden experienced by caregivers of patients with dementia [18]. However, a meta-analytic study concluded that the reliability of the instrument is validated across all caregivers of patients with different illnesses (e.g., cancer, dementia, physical illness, mental illness, etc.) for any population [19]. The scale consists of 22 items on a 5-point Likert scale ranging from never (0) to nearly always (4). The total score estimates the degree of burden that was recoded into 14–20 = little or no burden, 21–40 = mild to a moderate burden, 41–60 = moderate to a severe burden, 61–88 = severe burden. Cronbach’s alpha of Zarit burden scale was 0.87.

### Statistical analysis

Data were analyzed using SPSS version 20. Quantitative data were expressed as mean and standard deviation, while frequencies and percentages expressed the qualitative data.

Bivariate analysis was performed to explore the variables that were significantly associated with the stigma and burden of caregivers to be entered in the regression models. To identify the determinants of burden, affiliate, and associate stigma, three linear regression models were conducted. The independent variables in the models were either the significant variables in bivariate analysis or identified variables reported in the literature review. A significant difference was considered when the *p* value was less than 0.05.

## Results

Table 1 shows the socio-demographic characteristics of the caregivers and their mentally ill relatives. The mean age of caregivers was 45.1 ± 14.3 years, and females represented 60.7%. Most of the studied subjects were rural residents (86.1%) and approximately 70% were married. Those who were not working/housewives formed about 66.6%. More than half of caregivers (54.4%) were illiterates. Parents represented the main caregivers for about half of the mentally ill patients (48.9%) the mean age of the mentally ill patients was 32.7 ± 12.2 years. More than half of the patients (52.9%) were females. Nearly 50% were single and 39.1% were married. Illiterate patients formed approximately 43% while a small percentage (4%) had completed university education. Sixty percent of the patients were unemployed but financially supported by their own families, while only 2.4% were employed with maintained paid a fixed salary even in their illness.

**Table 1:** Socio-demographic characteristics of the study caregivers and their mentally ill patients, Assiut University Hospital, Egypt, 2017

Variables	Caregivers		Patients	
	No. (425)	%	No. (425)	%
**Age (years)**				
Range	21–85		5–75	
Mean ± SD	45.1 ± 14.3		32.7 ± 12.2	
**Gender**				
Male	167	39.3	200	47.1
Female	258	60.7	225	52.9
**Residence**				
Urban	59	13.9	59	13.9
Rural	366	86.1	366	86.1
**Marital status**				
Married	295	69.4	166	39.1
Single	54	12.7	206	48.5
Divorced	9	2.1	32	7.5
Widowed	67	15.8	21	4.9
**Educational status**				
Illiterate	231	54.4	182	42.8
Can read and write	37	8.7	32	7.5
Primary/preparatory	35	8.2	91	21.4
Secondary/technical/above average	90	21.2	103	24.2
University	32	7.5	17	4.0
**Caregivers occupation**				
Does not work/\housewife	283	66.6	–	–
Unskilled/skilled worker	66	15.5	–	–
Employee	25	5.9	–	–
Professional	9	2.1	–	–
Farmer	42	9.9	–	–
**Caregivers relationship with the patients**				
Parents	208	48.9	–	–
Brother/sister	103	24.2	–	–
Spouse (husband/wife)	48	11.3	–	–
Son/daughter	30	7.1	–	–
Others*	36	8.5	–	–
**Patients income**				
Employed and a fixed salary is paid even in his illness	–	–	10	2.4
Employed and no salary is paid in his illness	–	–	39	9.2
Unemployed and has a pension for his mother or father or exceptional	–	–	99	23.3
Unemployed and has no source of income, financially supported by his own family	–	–	257	60.5
Retired with a pension	–	–	1	0.2
Non-applicable (e.g., children)	–	–	19	4.5

*Others: (grandmother, uncle/aunt, nephew, cousin)

Table 2 describes the medical history of the studied mentally ill patients. The common mental illnesses among the studied patients were bipolar (48%), Schizophrenia/other related psychotic disorders (42.8%) and depression (6.8%). The duration of patient’s mental illness ranged from 1 to 40 years (mean ± SD = 7.93 ± 7.323). Approximately one third of patients had aggressive behavior (32.2%), while 15.5% had a suicidal history. Nearly 60% of patients were not admitted in psychiatric hospital in the past 6 months compared to 38% who were admitted for only one time. Psychiatric services were chosen as the first choice in seeking help by only 17.4% of the caregivers under study for their mentally ill relatives compared to 80% who sought care first from traditional healers.

**Table 2:** Medical history of the studied mentally ill patients, Assiut University Hospital, Egypt, 2017

Variables	No. (***n*** = 425)	%
**Mental illness diagnosis**		
Bipolar disorder	204	48.0
Schizophrenia and other related psychotic disorders	182	42.8
Depression	29	6.8
Child psychiatric disorders	10	2.4
**Mental illness duration (years)**		
Mean ± SE (Range)	7.93 ± 0.35^#^ (1–40)	
**Aggressive behavior (injuring/threatening to injure anyone)**		
Yes	137	32.2
No	288	67.8
**Suicidal history**		
Yes	66	15.5
No	359	84.5
**Frequency of hospital admission in the past 6 months**		
0	242	56.9
1	162	38.1
2	20	4.7
3	1	0.2
Mean ± SD	0.48±0.59	
**First sought care before hospital attendance**		
Traditional healer only	304	71.5
Psychiatrist from the start	74	17.4
Both traditional and non-psychiatric physician	37	8.7
Non-psychiatric physician only	10	2.4

^#^SD = 7.323

Table 3 shows that the mean scores of CPMI total scale, affective, cognitive, and behavioral subscales were 56.80 ± 7.99, 22.53 ± 3.23, 17.71 ± 3.84, 16.54 ± 2.85, respectively. The mean score of EMIC-Stigma scale was 13.81 ± 5.42. The mean score of Zarit burden scale was 55.20 ± 9.82. The mean value of ATMI total scale equalled 16.90 ± 4.22.

**Table 3:** Stigma, burden, and attitude toward mental illness of the caregivers, Assiut, Egypt, 2017

Variables	Range	Mean ± SD
**Affiliate Stigma Scale (CPMI)**		
Affective	10–28	22.53 ± 3.23
Cognitive	9–28	17.71 ± 3.84
Behavioral	8–27	16.54 ± 2.85
CPMI total scale	31–80	56.80 ± 7.99
**Associative stigma (EMIC-Stigma Scale)**	0–34	13.81 ± 5.42
**Zarit burden scale**	6–74	55.20 ± 9.82
**Caregiver’s attitude toward mental illness scale (ATMI)**		
Causes of mental illness	0–4	2.03 ± 1.19
Knowledge of people with mental illness	0–5	0.59 ± 1.03
Attitude toward people with mental illness	0–12	6.77 ± 2.74
Care and management of people with mental illness	2–12	7.49 ± 1.44
ATMI total scale	4–28	16.90 ± 4.22

**Fig. 1:**
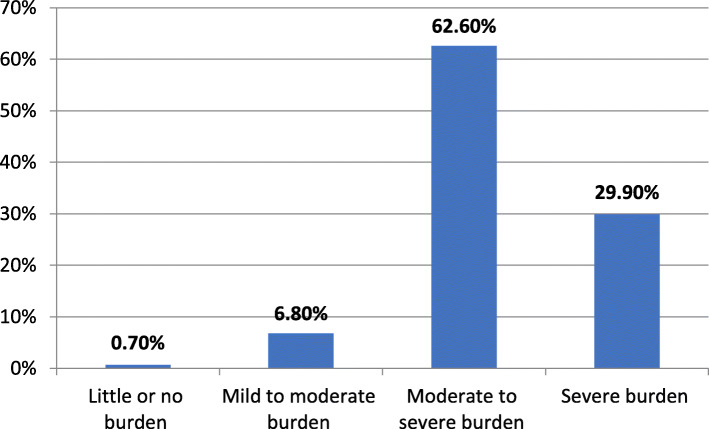
Level of burden among the studied caregivers assessed by Zarit burden scale

Most study caregivers (92.5%) suffered from moderate to severe and severe burden as shown in Fig. 1.

The significant correlates of the affiliate stigma of the studied caregivers as demonstrated in Table 4 were being parents of patients (*p* < 0.001) having higher associate stigma, and aggressive behavior of mentally ill patients, while Table 5 shows that being caregivers’ relatives other than parents, having higher affiliate stigma, having poor knowledge and negative attitude toward mental illness, and the aggressive behavior of mentally ill patients were the significant correlates for associate stigma of the study caregivers. The significant correlates for the perceived burden of the studied caregivers as demonstrated in Table 6 were the following caregivers’ criteria; being male, non-educated, having higher affiliate stigma, having higher associate stigma, having lower knowledge and negative attitude toward mental illness, seeking traditional healers and non-psychiatrists from the start, and caring after young mentally ill relatives.

**Table 4:** Correlates of affiliate stigma among the studied participants at Assiut University Hospital, 2017

Variables	Regression coefficient	***t*** value	***p*** value	95% CI
**Age of the caregiver**	0.013	0.452	0.651	− 0.045–0.071
**Caregiver gender** (female)	1.527	1.595	0.112	− 0.355–3.410
**Caregiver marital status** (single)	− 0.152	− 0.159	0.874	− 2.040–1.735
**Caregiver occupation** (does not work/housewife)	0.829	0.854	0.393	− 1.078–2.736
**Caregiver residence** (rural)	0.562	0.711	0.478	− 0.993–2.118
**Caregiver education** (illiterate/read and write)	0.470	0.694	0.488	− 0.862−1.801
**Caregiver relation** (parents)	4.529	5.208	**< 0.001**^**a**^	2.820–6.239
**Age of patient**	− 0.004	− 0.120	0.904	− 0.066–0.059
**Patient gender** (female)	− 0.310	− 0.550	0.583	− 1.416–0.797
**Patient marital status** (single)	0.748	1.202	0.230	− 0.475–1.972
A**ggressive behavior** (yes)	1.343	2.077	**0.038**^**a**^	0.072–2.615
**Suicide** (yes)	− 1.160	− 1.491	0.137	− 2.690–0.370
**Care first sought** (traditional healer or non-psychiatrist)	− 0.050	− 0.068	0.946	− 1.483–1.384
**Inpatient admission** (yes)	− 0.932	− 1.653	0.099	− 2.041–0.176
**Duration of current illness**	0.028	0.656	0.512	− 0.055–0.111
**Diagnosis of mental illness** (schizophrenia and other related psychotic disorders)	1.027	1.903	0.058	− 0.034–2.087
**Associate stigma (EMIC-Stigma Scale)**	0.793	14.508	**< 0.001**^**a**^	0.685–0.900
**ATMI Scale**	− 0.120	− 1.726	0.085	− 0.257–0.017

Adjusted linear regression model; *F* = 29.961, *p* < 0.001, adjusted *R*^2^ = 0.551

Reference groups: males, ever married, work, urban residence, educated at least primary education, other than parents, no injury, no suicide, psychiatrist, not inpatient admitted, other than schizophrenia

^a^Significant variable

**Table 5:** Correlates of associate stigma among the studied participants at Assiut University Hospital, Egypt, 2017

Variables	Regression coefficient	***t*** value	***p*** value	95 % CI
**Age of the caregiver**	0.011	0.486	0.627	− 0.032–0.053
**Caregiver gender** (female)	− 0.297	− 0.420	0.675	− 1.689–1.094
**Caregiver marital status** (single)	0.646	0.915	0.361	− 0.743–2.036
**Caregiver occupation** (does not work/housewife)	0.181	0.253	0.801	− 1.226–1.587
**Caregiver residence** (rural)	− 0.039	− 0.067	0.947	− 1.186–1.108
**Caregiver education** (illiterate/read and write)	− 0.221	− 0.442	0.659	− 1.202–0.761
**Caregiver relation** (parents)	− 1.815	− 2.767	**0.006**^**a**^	− 3.104–0.526
**Age of patient**	− 0.028	− 1.179	0.239	− 0.074–0.018
**Patient gender** (female)	− 0.154	− 0.370	0.711	− 0.969–0.662
**Patient marital status** (single)	− 0.563	− 1.229	0.220	− 1.465–0.338
**Aggressive behavior** (yes)	1.332	2.806	**0.005**^**a**^	0.399–2.265
**Suicide** (yes)	0.531	0.925	0.356	− 0.598–1.661
**Care first sought** (traditional healer or non-psychiatrist )	1.009	1.886	0.060	− 0.043–2.060
**Inpatient admission** (yes)	0.159	0.381	0.703	− 0.661–0.979
**Duration of current illness**	0.018	0.573	0.567	− 0.043–0.079
**Diagnosis of mental illness (**schizophrenia and other related psychotic disorders)	− 0.086	− 0.215	0.830	-0.871–0.699
**ATMI Scale**	− 0.158	− 3.107	**0.002**^**a**^	− 0.258 to − 0.058
**Affiliate stigma (CPMI-Stigma Scale)**	0.431	14.508	**< 0.001**^a^	0.372–0.489

Adjusted linear regression model; *F* = 21.980, *p* < 0.001, adjusted *R*^2^ = 0.471

Reference groups: males, ever married, work, urban, educated at least primary education, other than parents, no injury, no suicide, psychiatrist, not inpatient admitted, other than schizophrenia

^a^Significant variable

**Table 6:** Correlates of burden among the studied participants at Assiut University Hospital, Egypt, 2017

Variables	Regression coefficient	***t*** value	***p*** value	95 % CI
**Age of the caregiver**	− 0.079	− 1.958	0.051	− 0.158–0.000
**Caregiver gender** (female)	− 3.638	− 2.772	**0.006**	− 6.218 to − 1.058
**Caregiver marital status** (single)	0.205	0.156	0.876	− 2.375–2.784
**Caregiver occupation** (does not work/housewife)	1.809	1.347	0.179	− 0.830–4.448
**Caregiver residence** (rural)	− 0.282	− 0.261	0.794	− 2.411–1.847
**Caregiver education** (illiterate/read and write)	1.864	2.013	**0.045**^**a**^	0.043–3.685
**Caregiver relation** (parents)	1.419	1.157	0.248	− 0.992–3.830
**Age of patient**	− 0.136	− 3.003	**0.003**^**a**^	− 0.225 to − 0.047
**Patient gender** (female)	1.263	1.641	0.102	− 0.250–2.776
**Patient marital status** (Single)	− 1.087	− 1.260	0.208	− 2.782–0.609
**Patient income** (no constant income)	− 0.911	− 1.010	0.313	− 2.686–0.863
**Aggressive behavior** (yes)	0.301	0.339	0.734	− 1.444–2.047
**Suicide** (yes)	0.770	0.723	0.470	− 1.325–2.865
**Care first sought** (traditional healer or non-psychiatrist)	2.378	2.383	**0.018**^**a**^	0.416–4.341
**Inpatient admission** (yes)	0.382	0.494	0.622	− 1.137–1.900
**Duration of current illness**	0.104	1.801	0.072	− 0.010–0.217
**Diagnosis of mental illness (**schizophrenia and other related psychotic disorders)	0.272	0.368	0.713	− 1.183–1.727
**ATMI Scale**	− 0.221	− 2.315	**0.021**^**a**^	− 0.408 to − 0.033
**Affiliate stigma (CPMI-Stigma Scale)**	0.467	6.894	**< 0.001**^**a**^	0.334–0.601
**Associate stigma (EMIC-Stigma Scale)**	0.409	4.437	**< 0.001**^**a**^	0.228–0.590

Adjusted linear regression model; *F* = 18.099, *p* < 0.001, adjusted *R*^2^ = 0.446

Reference groups: males, ever married, work, urban, educated at least primary education, other than parents, constant income, no injury, no suicide, psychiatrist, not inpatient admitted, other than schizophrenia

^a^Significant variable

## Discussion

After the improvement of antipsychotics in the last two decades, there was a shift from hospital-based to community-based care. This shift added a high burden and stigma on family caregivers [6, 20]. In this study, the caregivers suffered from both affiliate and associate stigma. Affiliate stigma was highest for affective component followed by cognitive component and finally behavioral component. This hierarchy of different components is similar to that reported in previous studies from India among caregivers of patients with schizophrenia [15, 21]. This revealed that despite experiencing a high level of emotional distress and expecting negative reactions from others, caregivers of mentally ill patients do not neglect their mentally ill relatives and they would continue caring for them [21].

When the nature of the relationship between the caregivers and their patients was considered, our study showed that parents suffered from an affiliate stigma more than other patients’ relatives. Similarly, Indian [21] and Singaporean [22] parents of mentally ill patients experienced more affiliate stigma than other relatives. In fact, parents are more likely to blame themselves for contributing to their children’s illness, which may explain their more perception of affiliate stigma [21].

Compared to parents, being spouse and other patients’ relatives correlated with the associate stigma of the studied caregivers. This is consistent with a study in Sweden where spouses of mentally ill patients had more perception of associated stigma compared to other patients’ relatives [23]. Spouses may be exposed to greater stigma than parents because their ill relatives interfere with their social networks to a greater extent [23].

Behavioral manifestations of mental illness are important in shaping the caregiver stigma. The current study revealed that aggressive behavior of patients was correlated with the perception of the affiliate stigma among their caregivers. This is in concordance with a Chinese study where the severity of patients with schizophrenia’ positive symptoms, including aggressive symptoms was positively correlated with the perception of self-stigma among their caregivers [24]. A qualitative Indian study reported that patients with schizophrenia positive symptoms, particularly aggressive or disinhibited behavior in public, were linked to negative reactions towards caregivers and feelings of shame [25].

Similarly, aggressive behavior was correlated with the perception of associate stigma among the studied caregivers. This finding is consistent with a study in Flanders, which reported that the level of associate stigma among family members of patients with psychosis was significantly predicted by the burden of aggressive disruptions to family housemates of the patient with psychosis [26].

Poor knowledge and negative attitude toward mental illness did not affect the perception of self-stigma in the studied caregivers; however, it significantly increased their perception of associate stigma. This result is consistent with the study in India among caregivers of mentally ill patients where poor knowledge about mental illness causation, signs and symptoms, and leaning toward modern methods of treatment were positively associated with their perception of associate stigma [4].

The perception of affiliate stigma was significantly correlated with associate stigma in the studied subjects. This can be explained by the internalization of self-stigma by the caregivers of mentally ill relatives as a consequence of their affection by public stereotype (public stigma), which they integrate into their self-concept [27].

The studied caregivers suffered a high burden; the mean score of Zarit burden scale was 55.20 ± 9.82. This rate is higher than that detected among caregivers of bipolar disorder patients in Brazil (32.0 ± 19) and in caregivers of patients with schizophrenia in Iran (51.73 ± 18.23) [28, 29]. In contrast, the mean burden score was higher (64.51 ± 12.97) among caregivers of patients with schizophrenia in South America [30]. However, the percentage of those experienced moderate to severe or severe burden was similar to the current study (92.2%) [30].

In this study, the male gender of the caregiver was a correlate of higher burden perception in linear regression. A systematic review of gender differences in caregiving among family caregivers of people with mental illnesses concluded that gender difference in caregiving is confusing and inconsistent. Caregiver gender explains only a minor proportion of the variance in the caregiving burden [31].

The educational level of the caregivers is a variable that can modulate the degree of burden experienced; non-educated caregivers (illiterates/read and write) significantly experienced more burden in the current study. This finding is consistent with different studies in South America and Cyprus where the caregivers with higher levels of education had less burden [30, 32].

Concerning the association between caregivers’ burden and patients’ age, caring after a young mentally ill relative was a predictor of the increasing burden of the studied subjects. Young patients may have a more severe form of mental illness and/or lack adequate vocational and independent living skills which increases their caregivers’ burden [33].

Having higher affiliate stigma was associated with a significant increased level of burden of the studied subjects. This result conformes to a similar American study where there was a significant correlation between caregiving burden and affiliate stigma [34]. Similarly, having higher associate stigma was significantly associated with an increasing level of burden of the studied caregivers. This result is in concordance with a cross-sectional survey among family members of people with a mental illness in the USA where the perception of associate stigma was a significant predictor of greater psychological distress [35].

The diagnosed type of mental illness was not associated with caregivers’ burden or stigma. This finding is consistent with different studies among Polish, German, Austrian, Euro-American, and Sweden caregivers, where the overall caregivers’ burden and stigma were independent of the type of mental illness [23, 36–38].

The pattern of seeking care for mental illness can modulate the level of burden experienced by the caregivers. Seeking traditional healers and non-psychiatric care from the start was a significant predictor of the higher level of burden of the studied subjects. This can be interpreted by the fact that seeking care through traditional healers and non-psychiatrists leads to delayed treatment of the patients’ illness, so more problematic behavior and hence more burdens can be perceived [39].

Unfortunately, traditional healers represent the highest percentage of the first consulted care providers in mental illness in Egypt [12, 40, 41]. Most studied caregivers (80%) sought advice for the first time from traditional healers for the care of their mentally ill relatives. This finding is concordant with the results of other Egyptian studies. In Al Minia University Hospital, 86.8% of patients were treated by non-psychiatric medical and traditional services before they sought psychiatric care [12]. In addition, 60% of outpatients attending Ain Shams University Psychiatric Clinic in Cairo have been traditional healers before coming to psychiatrists [40]. Nearly three quarters (77.5%) of patients with schizophrenia in Ismailia have attended traditional treatment, and 59% of them sought it as a first treatment choice [41].

### Limitations of the study

The study is limited by its cross-sectional nature, which inhibits a complete understanding of the causal relationship between determinants and caregiving outcomes. Due to the nature of the mental illness, the researchers applied a hospital-based study and used a purposive sampling technique, which limits the generalization of the findings to all family caregivers of the mentally ill in the community.

## Conclusions and recommendations

This study is one of the earliest studies in Upper Egypt that targeted family caregivers of mentally ill patients. Unfortunately, the studied caregivers’ suffered from stigma and a high level of burden. Mental health institutions should provide psycho-educational programs to all family caregivers of mentally ill patients to improve their knowledge and attitude toward mental illness and enable them to cope with the aggressive behavior of their mentally ill relatives. A future national representative study is recommended to explore the stigma and burden perception among those caregivers and to compare the situation between different Egyptian areas. Also, a qualitative study is recommended to explore details of the caregivers’ experiences of stigma and caregiving burden.

## Data Availability

The data sets generated and analyzed during the current study are available from the corresponding author on reasonable request.

## Abbreviations

WHO: World Health Organization
DSM-5: Diagnostic and Statistical Manual of Mental Disorders-Fifth Edition
CPMI: Stigma scale for Caregivers of People with Mental Illness
EMIC-Stigma scale: Explanatory Model Interview Catalogue-stigma scale
ATMIQ: Attitude Toward Mental Illness Questionnaire
USA: United States of America
